# Plasma Metabolomics for Discovery of Early Metabolic Markers of Prostate Cancer Based on Ultra-High-Performance Liquid Chromatography-High Resolution Mass Spectrometry

**DOI:** 10.3390/cancers13133140

**Published:** 2021-06-23

**Authors:** Xiangping Lin, Lucie Lécuyer, Xinyu Liu, Mohamed N. Triba, Mélanie Deschasaux-Tanguy, Aïcha Demidem, Zhicheng Liu, Tony Palama, Adrien Rossary, Marie-Paule Vasson, Serge Hercberg, Pilar Galan, Philippe Savarin, Guowang Xu, Mathilde Touvier

**Affiliations:** 1 Sorbonne Paris Nord University, Chemistry Structures Properties of Biomaterials and Therapeutic Agents Laboratory (CSPBAT), Nanomédecine Biomarqueurs Détection Team (NBD), The National Center for Scientific Research (CNRS), UMR 7244, 74 Rue Marcel Cachin, CEDEX, 93017 Bobigny, France; xiangping.lin@outlook.com (X.L.); mohamed.triba@univ-paris13.fr (M.N.T.); tony.palama@univ-paris13.fr (T.P.); 2 CAS Key Laboratory of Separation Science for Analytical Chemistry, Dalian Institute of Chemical Physics, Chinese Academy of Sciences, Dalian 116023, China; liuxy2012@dicp.ac.cn (X.L.); xugw@dicp.ac.cn (G.X.); 3 Sorbonne Paris Nord University, Nutritional Epidemiology Research Team (EREN), Epidemiology and Statistics Research Center Inserm U1153, Inrae U1125, Cnam, University of Paris (CRESS), 74 Rue Marcel Cachin, CEDEX, 93017 Bobigny, France; l.lecuyer@eren.smbh.univ-paris13.fr (L.L.); hercberg@uren.smbh.univ-paris13.fr (S.H.); galan@uren.smbh.univ-paris13.fr (P.G.); m.touvier@eren.smbh.univ-paris13.fr (M.T.); 4 Cellular Micro-Environment, Immunomodulation and Nutrition (ECREIN), Human Nutrition Unit (UNH), Clermont Auvergne University, INRAE, UMR 1019, CRNH Auvergne, 63000 Clermont-Ferrand, France; aicha.demidem@inra.fr (A.D.); Adrien.rossary@uca.fr (A.R.); m-paule.vasson@uca.fr (M.-P.V.); 5 School of Pharmacy, Anhui Medical University, Hefei 230032, China; liuzhicheng@ahmu.edu.cn; 6 Anticancer Center Jean-Perrin, CHU Clermont-Ferrand, CEDEX, 63011 Clermont-Ferrand, France

**Keywords:** metabolomics, LC-MS, multivariate analysis, prostate cancer, biomarkers

## Abstract

**Simple Summary:**

Correct identification of subjects at high risk is critical in the prevention and early screening of prostate cancer (PCa). Analysis of metabolites in biofluids has shown to be a promising method to identify novel PCa biomarkers. To identify potential biomarkers of PCa, we conducted metabolic profiling of pre-diagnosis plasma metabolite profiles from a large prospective male cohort (*n* = 418), which included 146 males who developed PCa during a 13-year follow-up and 272 matched controls to investigate the relationship with long-term PCa risk. We show metabolite profiles discriminate males who subsequently developed PCa during the follow-up from matched controls with a high degree of accuracy (AU-ROC 0.92) and highlight 10 metabolites associated with a high risk of PCa. These results suggest that the dysregulation of amino acids and sphingolipid metabolism is associated with future risk of PCa.

**Abstract:**

Background: The prevention and early screening of PCa is highly dependent on the identification of new biomarkers. In this study, we investigated whether plasma metabolic profiles from healthy males provide novel early biomarkers associated with future risk of PCa. Methods: Using the *Supplémentation en Vitamines et Minéraux Antioxydants* (SU.VI.MAX) cohort, we identified plasma samples collected from 146 PCa cases up to 13 years prior to diagnosis and 272 matched controls. Plasma metabolic profiles were characterized using ultra-high-performance liquid chromatography-high resolution mass spectrometry (UHPLC-HRMS). Results: Orthogonal partial least squares discriminant analysis (OPLS-DA) discriminated PCa cases from controls, with a median area under the receiver operating characteristic curve (AU-ROC) of 0.92 using a 1000-time repeated random sub-sampling validation. Sparse Partial Least Squares Discriminant Analysis (sPLS-DA) identified the top 10 most important metabolites (*p* < 0.001) discriminating PCa cases from controls. Among them, phosphate, ethyl oleate, eicosadienoic acid were higher in individuals that developed PCa than in the controls during the follow-up. In contrast, 2-hydroxyadenine, sphinganine, L-glutamic acid, serotonin, 7-keto cholesterol, tiglyl carnitine, and sphingosine were lower. Conclusion: Our results support the dysregulation of amino acids and sphingolipid metabolism during the development of PCa. After validation in an independent cohort, these signatures may promote the development of new prevention and screening strategies to identify males at future risk of PCa.

## 1. Introduction

Prostate cancer (PCa) is the second most commonly diagnosed cancer and the second leading cause of cancer death (7.1% for incidence) among males [1]. Currently, there is no single definitive test to identify PCa in this particular population [2]. The Prostate-Specific Antigen (PSA) test and digital rectal examination are the methods used for PCa screening; for definitive diagnosis, prostate biopsy and supplementary imaging are required [3].

Measurement of the PSA is relatively easy in large-scale populations; however, the usefulness of the PSA test is still under debate due to the poor specificity for detecting cancer and for differentiating indolent cancers from high-risk ones [2]. The limited specificity of the PSA test results in overdiagnosis (unnecessary prostate biopsies) and consequently, the overtreatment of subjects with a low-potential malignant tumor, or with a low potential for morbidity or death if left untreated [4,5]. 

The identification of novel biomarkers is crucial in the management process of the disease. Although extensive efforts in biomarker discovery during the last few decades have been undertaken, including screening for genetic risk factors and biomarkers [6,7], sensitive and specific biomarkers are still urgently needed in the prevention, early screening, monitoring, and clinical management of subjects at risk of developing PCa [8,9,10,11]. Different approaches are now studied in this aim, for example, the description of the tumor microenvironment [12]. Among these approaches, new omics-based biomarkers (genomics, transcriptomics, proteomics, or metabolomics) have been validated and seem to enhance the diagnosis or the prognostic of PCa (for a review, see [13]).

Among those, metabolomics methods that provide a high-dimensional characterization of low molecular weight biochemicals (metabolites) have shown to be a promising and powerful tool to identify novel PCa biomarkers in biofluids [14,15,16,17,18,19,20,21,22,23]. New advances have been found to describe metabolic alterations that describe tumor growth [24]. The metabolic landscape of tissue and urine was characterized in other studies (for a review, see [25]), and new statistical approaches have been tested for the metabolic profiling of prostate tissue and serum samples [20]. Untargeted metabolomics is a “hypothesis-generating discovery strategy” that compares different groups of samples (e.g., cancer vs. controls) [26], which is a promising approach to identify novel metabolic markers. This strategy has been applied recently in PCa [19,27]. Thus, the combination of ultra-high-performance liquid chromatography with high-resolution mass spectrometry (UHPLC-HRMS) and epidemiological approaches may open new perspectives in PCa research, enabling the identification of novel biomarkers for evaluating the future risk of PCa, and the investigation of the etiology of PCa [28,29,30]. To the best of our knowledge, there is still a very limited number of studies that have investigated the association between pre-diagnostic levels of plasma metabolites and the risk of PCa incidence [31]. 

In the present study, a prospective nested case–control study was established using the *Supplémentation en Vitamines et Minéraux Antioxydants* (SU.VI.MAX) cohort [32,33]. The study design included identification of baseline plasma samples from 146 individuals who developed PCa within a 13-year follow-up and 272 matched controls. Plasma samples were analyzed using UHPLC-HRMS to investigate whether plasma untargeted metabolic profiles could identify new early metabolic markers, if any, associated with the risk of developing PCa within the following decade.

## 2. Materials and Methods

### 2.1. Population Study

Participants in the present study were selected from the SU.VI.MAX (*Supplémentation en Vitamines et Minéraux Antioxydants*) prospective cohort (clinicaltrials.gov; NCT00272428) [32,33], which included 146 participants diagnosed with PCa during the 13-year follow-up, and 272 matched controls. The SU.VI.MAX cohort was initially designed as a double-blind placebo-controlled trial; the aim was to investigate the influence of daily supplementation with nutritional doses of antioxidants on the incidence of cardiovascular diseases and cancers. Briefly, a total of 13 017 participants were enrolled between 1994 and 1995 for an 8-year intervention trial and were followed for outcomes until September 2007. The study was conducted according to the guidelines of the Declaration of Helsinki and approved by the Ethics Committee for Studies with Human Subjects of Paris-Cochin Hospital (CCPPRB 706/2364) and the ‘Commission Nationale de l’Informatique et des Libertés’ (CNIL 334641/907094) [32,33].

#### 2.1.1. Baseline Data Collection

At enrollment, all participants underwent a clinical examination that included anthropometric measurements and a blood draw, occurring after a 12 h fasting period. Information on socio-demographics, smoking habits, physical activity, medication use, and health status were collected through self-administered questionnaires. A 35 mL venous blood sample was collected in sodium heparin Vacutainer tubes (Becton Dickinson, Rungis, France) from all fasting participants. After centrifugation at 4 °C, plasma aliquots were immediately prepared and stored frozen at −20 °C for up to 2 days and then stored in liquid nitrogen.

#### 2.1.2. Case Ascertainment

Health events were self-reported by the participants in regular follow-up questionnaires. All relevant medical information and pathological reports were gathered through participants, physicians and/or hospitals and subsequently reviewed by an independent physician expert committee. Validated cases were classified according to the International Chronic Diseases Classification, 10th Revision (ICD-10) [34].

#### 2.1.3. Nested Case–Control Study

All participants with a first incident invasive prostate cancer, diagnosed with 1 year of their inclusion in the SU.VI.MAX cohort in 1994–1995 and September 2007 were included in this nested case–control study (*n* = 146). Incident prostate cancers diagnosed during the first year of follow-up were excluded to avoid reverse-causality bias and guarantee the prospective design. The method for control selection was density sampling, i.e., every time a case was diagnosed, two controls were selected from other members of the cohort who, at that time, were not diagnosed with PCa. In this study, for each case, two controls (or one if sample is limited) were randomly selected and matched for baseline age (45–49 years/50–54 years/55–59 years/>60 years), body mass index (BMI) (underweight, normal weight and overweight/obese), intervention group of the initial SU.VI.MAX trial (placebo/supplemented), smoking status (current smokers and non-smokers), and season of blood draw (a priori-defined periods: October–November/December–January–February/March–April–May). 

### 2.2. UHPLC-HRMS Metabolomic Analysis

Plasma samples were randomized, balanced based upon case and control, and thawed on ice. Aliquots of the first 160 plasma samples were pooled as QC samples. For deproteinization and metabolites extraction, 150 μL of plasma was mixed with 600 μL ice-cold methanol containing internal standards (ISs) (Supplemental Table S1), after vortexed for 2 min, centrifuged for 10 min, at 16,000× *g*, at 4 °C. Three aliquots with 50 μL supernatant each were lyophilized (prepared for positive mode analysis, for negative mode analysis, and for backup), then stored at −80 °C before analysis. For quality control during sample preparation, a QC sample was prepared with every 10 plasma samples. Before analysis, the lyophilized supernatant was re-dissolved in 50 μL acetonitrile/water (1:3, *v*/*v*) solvent, after being vortexed 2 min and centrifuged 10 min at 14,000 rpm, at 10 °C. For metabolomic analysis, 5 μL of re-dissolved supernatant was injected, which was performed on an ACQUITY Ultra Performance Liquid Chromatography (UHPLC, Waters Corporation, Manchester, UK), coupled with a Q Exactive HF Orbitrap MS system (Thermo Fisher Scientific, Rockford, IL, USA). UHPLC column temperature and automatic sampler temperature were set at 60 °C and 10 °C, respectively. 

Before run study samples, the Q-Exactive HF MS ion source, ion transfer tube were cleaned, the MS was evaluated, and calibrated; several blank samples and 10 QC samples were tested to confirm system suitability. Metabolomic analysis was performed in electrospray positive ion (ESI+) mode with reversed-phase ACQUITY UPLC BEH C8 (2.1 × 50 mm, 1.7 μm, Waters, Milford, MA, USA) column for separation of weakly polar compounds such as carnitine and lipids. Mobile phases include water containing 0.1% formic acid (A) and acetonitrile containing 0.1% formic acid (B). The flow rate was 0.40 mL/min and the total run time was 12 min. The elution program started with 5% B and was held for 0.5 min, then linearly increased to 40% B at 2 min and increased to 100% B at 8 min, maintained 2 min, then went back to 5% B in 0.1 min and kept for 1.9 min for post-equilibrium. The resolutions of full-scan MS and ddMS2 were set at 120,000 FWHM (at *m*/*z* 200, at 3 Hz) and 60,000 FWHM (at *m*/*z* 200, at 7 Hz), respectively. The mass accuracy is < 1 ppm RMS (root mean square) error using internal calibration, and < 3 ppm RMS error using external calibration. The scan range was set from 70 to 1050 *m*/*z*; the automatic gain control (AGC) target and maximum injection time in full-scan MS settings were 3 × 10^6^ and 100 ms, while their values were 1 × 10^5^ and 50 ms in ddMS2 settings. The TopN (N, the number of topmost abundant ions for fragmentation) was set to 10, and collision energy (NCE) was set to 15, 30, and 45. A heated ESI source was used. The spray voltage was set at 3.5 kV. The capillary temperature and aux gas heater temperature were set at 300 and 350 °C, respectively. Sheath gas and aux gas flow rate were set at 45 and 10 (in arbitrary units), respectively. The S-lens rf level was 50. Additional technical details are given in the Supplemental Data and Methods. 

Metabolite detection and peak integration were first optimized using TraceFinder (version 4.1, Thermo Fisher Scientific, Rockford, IL, USA); the full dataset was extracted using Compound Discoverer (version 3.0, Thermo Fisher Scientific, Rockford, IL, USA) for peak detection, integration, and identification. Metabolite identification was performed by comparing high-resolution accurate *m*/*z* and retention time to the in-house standard databases in the same laboratory; if the ddMS2 information is available for the precursor in the QC samples, then MSMS data is compared with the help of Xcalibur software (version 4.2, Thermo Fisher Scientific, Rockford, IL, USA), Compound Discoverer software (version 3.0, Thermo Fisher Scientific, Rockford, IL, USA), or fragmentation information in the Human Metabolome Database (HMDB) (version 4.0). 

The missing values were estimated using the eigenvector method in a cross-validation process [35,36]. Metabolomic data were normalized by using probabilistic quotient normalization [37] to account for dilution of complex biological mixtures, centering, unit variance scaling, and generalized logarithm transformed [38]. Following the logarithmic transformation, a normality test was performed for each variable. Principal component analysis (PCA) was used as a quality assessment tool to verify clustering of intra-study QC samples before data analysis (Supplemental Figure S3). Before further statistical analysis, a PCA was performed on QC samples as suggested by Chan et al. [39]. Variability in QC samples is experimental and is uncorrelated with biological variability of interest. We built a 2-component PCA model on QC samples to summarize the structured part of this experimental variability. This variability was then removed from the others samples by performing an orthogonal projection relatively to the PCA scores. 

### 2.3. Statistical Analysis

Comparison of participants’ baseline population characteristics, including clinical factors for cases and controls, were first assessed using the conditional logistic regression models; *p*-values for clinical factors were calculated with the conditional logistic regression model.

Orthogonal partial least squares discriminant analysis (OPLS-DA) was used to identify metabolic differences between cases and controls. OPLS-DA was performed using in-house OPLS script based on Trygg and Wold method [40]; analyses were performed with MATLAB^®^ (R2016b for macOS; Mathworks, Natick, Massachusetts, USA). Quality parameters of the models, the explained variance (R2Y), and the predictability of the model (Q2Y) were determined. Q2Y was calculated by a 7-fold cross-validation and confirmed by exploring the impact of permutations in the dataset rows [41]. 

To assess the OPLS-DA model accuracy, the AU-ROC [41,42] was determined using a repeated random sub-sampling validation (also referred to as Monte Carlo cross-validation), which splits the dataset randomly into discovery and validation sets. In our study, for each random resampling, a discovery cohort (randomly selected 70% of all participants, with prostate cancer cases: *n* = 102/controls: *n* = 190) was used to establish an OPLS-DA model; the model fit was then evaluated by predicting case and control status classification in the corresponding validation cohort (remaining of all participants, with prostate cancer cases: *n* = 44/controls: *n* = 82), and an AU-ROC for each prediction was calculated. This process was repeated 1000 times.

GraphPad Prism 8 (GraphPad Software, Inc., 2018, La Jolla, CA, USA) was used to plot the ROC curve and the box-and-whisker plots (Tukey, 1977) [43]; ROC curve was computed using DeLong et al. (1988) [44], and the Youden index J, is defined as: J = max (sensitivity c + specificity c − 1), where c ranges over all possible criterion values [45]. Equal weight was given to sensitivity and specificity. For the box-and-whisker plot, outside and far-out values were identified according to the original definitions of Tukey [43].

For variable selection, sparse partial least squares discriminant analysis (sPLS-DA) [46] was performed using MetaboAnalyst 4.0 [47]; the number of components was fixed at 5 in the model, and the number of variables in each component was fixed at 15, with repeated random resampling. sPLS-DA allows the selection of the most predictive or discriminative variables in the data that help classify the samples; the feature selection is based on least absolute shrinkage and selection operator (LASSO) penalization on the pair of loading vectors [46]. 

To show relative changes of selected metabolites between the cancer and control groups, *t*-test with Bonferroni adjustment was carried out by using Multiple Experiment Viewer (MeV_4_9_0_r2731_win) [48]. To investigate possible relationship between baseline metabolite and risk of PCa during follow-up, binomial logistic regression analyses were performed, the odds ratio (OR) and p-values for coefficients of logistic regression model were computed with “glm” function [49] by using RStudio (version 1.3.1056, R version 4.0.2).

## 3. Results

### 3.1. Characteristics of PCa Cases and Matched Controls

The baseline characteristics of PCa cases (*n* = 146) and matched controls (*n* = 272) are summarized in Table 1. The mean age at PCa diagnosis was 63 years old; the average time between blood collection and diagnosis was 8.3 years. Compared to the matched controls, participants who developed PCa during the follow-up had a higher baseline level of PSA and were more likely to have a family history of PCa.

### 3.2. Discrimination of PCa Cases from Controls Using OPLS-DA Model

**Figure 1:**
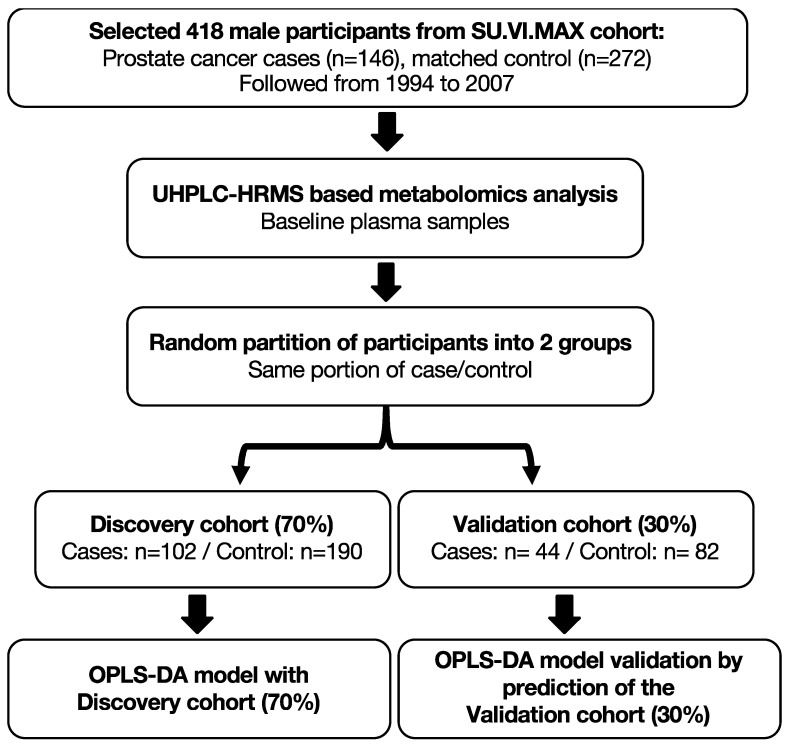
Simplified scheme of the study, OPLS-DA model, and validation. First, plasma samples from 418 male participants enrolled in the SU.VI.MAX cohort, which included prostate cancer cases (*n* = 146) and matched control (*n* = 272) were randomly partitioned into a discovery cohort (randomly selected 70% of all samples, with cases: *n* = 102/control: *n* = 190) and validation cohort (remaining of the cohort, 30% of all samples, with PCa cases: *n* = 44/control: *n* = 82), with an equal proportion of case/control. Then, an OPLS-DA model for classification of prostate cancer cases and matched controls was fit using the discovery cohort, the OPLS-DA model was then validated by predicting samples in the corresponding validation cohort, and an AU-ROC for prediction was calculated.

A total of 214 identified metabolites (MSI level 1) [50] were detected in the plasma samples. In the discovery cohort (70% randomly selected participants, with PCa cases: *n* = 102/controls: *n* = 190), an OPLS-DA model for the classification of PCa cases and matched controls was investigated (Figure 1). The model had five components (1 predictive component and four orthogonal components), suggesting a good model fit (R2: 0.73) and fairly good predictive power (Q2cum: 0.48). Supplemental Figure S1 includes the model score plots and shows the projection and classification of samples in the OPLS-DA model.

**Figure 2:**
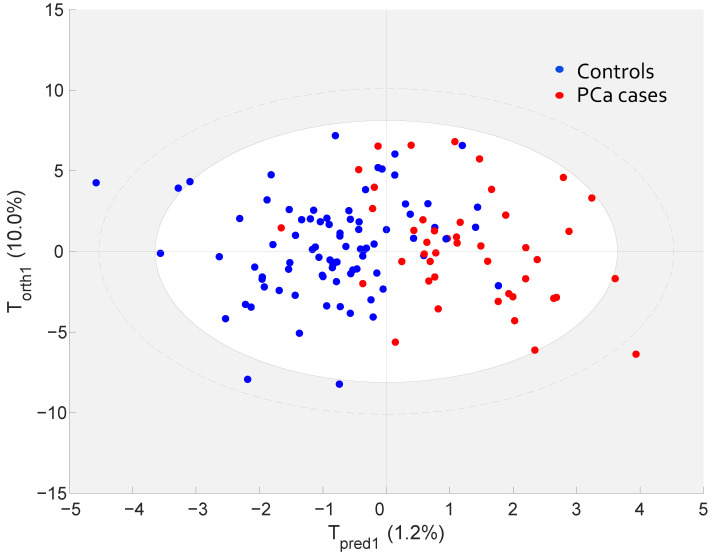
Projection of validation cohort samples using discovery cohort OPLS-DA model. Validation cohort, PCa cases (*n* = 44; red circle), matched controls (*n* = 82; blue circle). Corresponding AUC: 0.92 (sensitivity: 86.36%; specificity: 86.59%), 95% confidence interval (0.87, 0.97), *p* value: < 0.0001.

The OPLS-DA model was validated by the prediction of samples in the validation cohort (remaining 30% of the cohort, with PCa cases: *n* = 44/controls: *n* = 82) (Figure 1). Figure 2 shows the projection of the validation cohort samples into the OPLS-DA model from the discovery cohort. There is a clear discrimination of participants who developed PCa during the follow-up from controls, with an AU-ROC (Supplemental Figure S2): 0.92 (sensitivity: 86.36%; specificity: 86.59%), 95% confidence interval (0.87, 0.97), *p*-value < 0.0001. Thus, our results suggest the OPLS-DA model provides high degrees of accuracy for discriminating PCa cases from controls.

**Figure 3:**
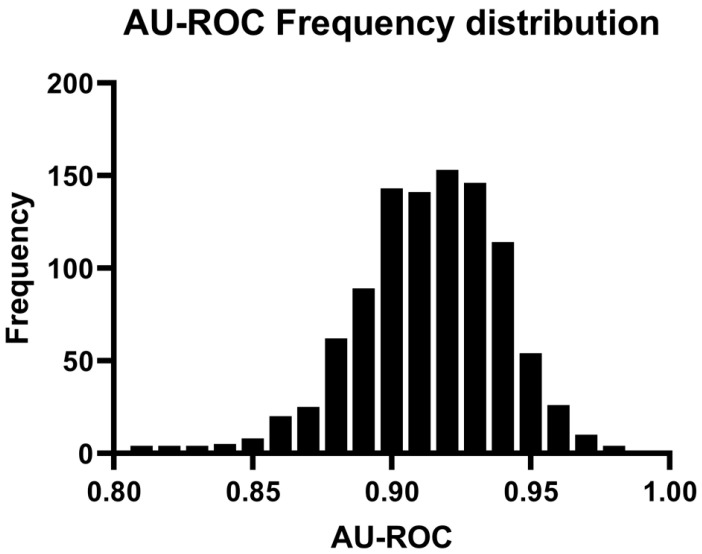
AU-ROC distribution for validation cohort during a 1000-time repeated random sub-sampling validation (median: 0.92, min: 0.81, max: 0.98). For each resampling, a discovery cohort (randomly selected 70% of all samples, with PCa cases: *n* = 102/controls: *n* = 190) was used to establish an OPLS-DA model, the model was then validated by predicting samples in the corresponding validation cohort (remainder of all samples, with PCa cases: *n* = 44/controls: *n* = 82), and an AU-ROC for each prediction was calculated. AU-ROC, Area under the receiver operating characteristic curve.

The OPLS-DA model was validated further by a 1000-fold repeated random sub-sampling validation. Figure 3 shows the AU-ROC distribution for each prediction during a 1000-fold repeated random sub-sampling validation, with a median value: 0.92, minimum value: 0.81, maximum value: 0.98. These results confirm the very good performance of our OPLS-DA model in the discrimination of participants who subsequently developed PCa during the follow-up from controls.

### 3.3. Identification of Metabolites Associated with Risk of Developing PCa

To highlight the metabolites which best discriminate PCa cases from controls, sPLS-DA was performed. sPLS-DA is a LASSO penalization-based variable selection method, which allows the selection of the most predictive or discriminative variables in the data that contributes to sample classification [46]. An error rate of 18% was finally obtained. Metabolites were ranked based upon their contribution to the discrimination of participants who developed PCa, and the top 10 metabolites were characterized further (Supplemental Table S2).

**Figure 4:**
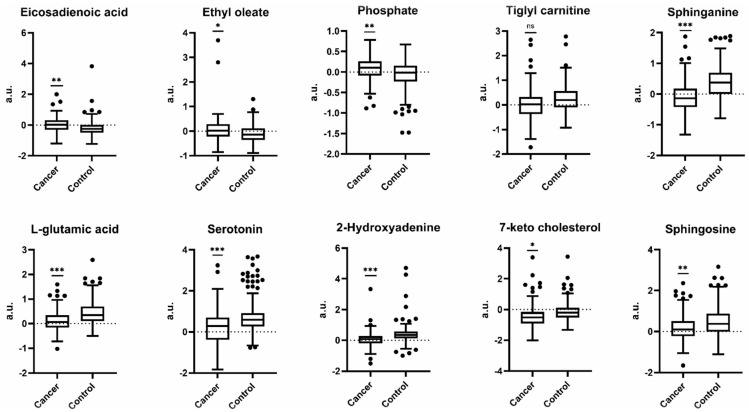
Box plots of peak areas for the 10 discriminants metabolites in participants who developed PCa during the follow-up and matched controls. Eicosadienoic acid, ethyl oleate, and phosphate were relatively higher in PCa group than in controls; on the contrary, L-glutamic acid, 2-hydroxyadenine, 7-keto cholesterol, tiglyl carnitine, serotonin, sphinganine, and sphingosine were relatively lower in PCa group than in controls. Sparse partial least squares discriminant analysis (sPLS-DA) was used to identify the top 10 most important metabolites discriminating PCa cases (*n* = 146) from controls (*n* = 272). Significance was determined by p-value with Bonferroni adjustment (Supplemental Table S2.): * *p* < 0.05; ** *p* < 0.01; *** *p* < 0.001; ns, not significant. The y-axis represents peak areas after removing variability in QC samples, probabilistic quotient normalized, centering, unit variance scaling, and generalized logarithm transformed. a.u.: arbitrary unit.

**Figure 5:**
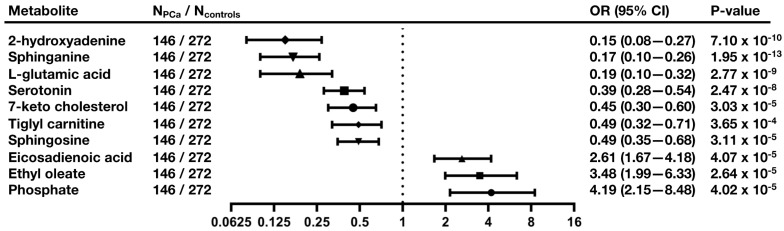
Relationship between baseline metabolites and risk of developing PCa during the follow-up. The x-axis represented log2 transformed scale. *p*-value from binomial logistic regression models, OR, odds ratio; CI, confidence interval.

For these 10 metabolites, differences for the PCa and control groups are provided in Figure 4. Among them, phosphate (OR = 4.19; 95% CI: 2.15–8.48; *p* = 4.02 × 10^−5^), ethyl oleate (OR = 3.48; 95% CI: 1.99–6.33; *p* = 2.64 × 10^−5^), and eicosadienoic acid (OR = 2.61; 95% CI: 1.67–4.18; *p* = 4.07 × 10^−5^) were positively associated with risk of developing PCa during the follow-up period (Figure 5 and supplemental Table S2). In contrast, inverse associations with risk of developing PCa during the follow-up were observed for 2-hydroxyadenine (OR = 0.15; 95% CI: 0.08–0.27; *p* = 7.10 × 10^−10^), sphinganine (OR = 0.17; 95% CI: 0.10–0.26; *p* = 1.95 × 10^−13^), L-glutamic acid (OR= 0.19; 95% CI: 0.10–0.32; *p* = 2.77 × 10^−9^), serotonin (OR= 0.39; 95% CI: 0.28–0.54; *p* = 2.47 × 10^−8^), 7-keto cholesterol (OR = 0.45; 95% CI: 0.30–0.60; *p* = 3.03 × 10^−5^), tiglyl carnitine (OR = 0.49; 95% CI: 0.32–0.71; *p* = 3.65 × 10^−4^), and sphingosine (OR = 0.49; 95% CI: 0.35–0.68; *p* = 3.11 × 10^−5^) (Figure 5).

## 4. Discussion and Conclusions

In the present study, we characterized plasma metabolic profiles collected from healthy males prior to PCa diagnosis and matched controls. Comparison of plasma samples from participants who developed PCa at follow-up identified 10 metabolites (Figure 4 and Figure 5, Supplemental Table S2) that may be useful to identify males at higher risk of developing PCa. It should be noted at the beginning that one limitation of our study is that the storage (less than 2 days for all the samples at −20 °C) could influence the metabolite levels in the sample [51].

**Figure 6:**
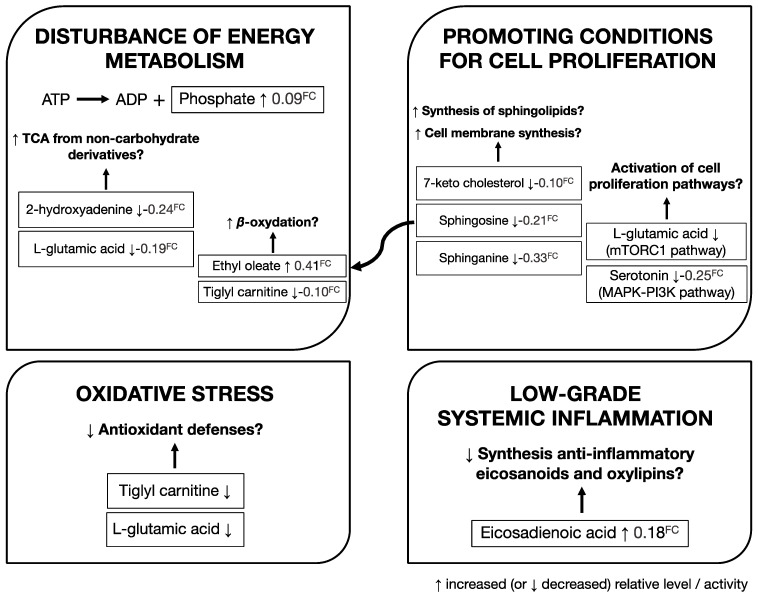
A model for metabolic changes during development of prostate cancer. Among these 10 important metabolites (*p* < 0.001) in the discrimination of PCa cases from controls, the majority are related to amino acids and sphingolipid metabolism and participated in energy metabolism, cell proliferation, oxidative stress, and inflammation. Our results suggest possible changes or perturbations in these physiological processes in males who subsequently developed PCa during the follow-up. FC: fold change.

Among these 10 metabolites, the majority are related to amino acids and sphingolipid metabolism, including metabolites that contribute to energy metabolism, cell proliferation, oxidative stress, and inflammation (Figure 6). Our results suggest possible changes or perturbations in these physiological processes in males at risk for PCa.

Decreased plasma glutamic acid levels were observed in participants who subsequently developed PCa during follow-up (Figure 6). Plasma glutamic acid is a compound derived from glutamine (Gln), an ammonia molecule NH_3_ linked to glutamate, which allows the transport of the nitrogen produced at the periphery to the liver to be eliminated by the urea cycle. This result is in concordance with a previous study on PCa risk using NMR-based metabolomics performed by our group [52], which showed that plasma glutamine level was negatively correlated with urea level. The results of these two studies (NMR and MS based studies) suggest a dysfunction of the urea cycle in males who subsequently developed PCa during follow-up, leading to a decrease in urea production, an accumulation of glutamine upstream, and a drop in glutamic acid. Gln is also a regulator of many pathways, including the mTOR pathway, a major pathway for cell proliferation. The relation between nitrogen providing from Gln and the activation of the mTOR pathway has previously been reported [53]. Instead, the dietary supplementation of alpha ketoglutarate activates mTOR signaling by improving energy metabolism [54]. The oxidation of the carbon skeleton of Gln (glutamic acid) in the mitochondria is a major source of energy for proliferating cells, including tumor cell lines. Finally, glutamic acid participates in antioxidant defense as a constituent of glutathione (major cellular antioxidant) [55]. 

Furthermore, in the NMR-based study [52], an increase in histidine level was reported in men who developed PCa during the follow-up. This observation is consistent with the decrease in glutamate observed in our study, in view of the metabolic links between these two amino acids. The dysregulation of this pathway has been observed in another cohort study [22] and other types of cancers as well, such as in breast cancer [56] and in hepatocellular carcinoma (HCC) [57]. 

2-Hydroxyadenine is a hydrogenated derivative of guanine. The observed decreased plasma levels of 2-hydroxyadenine could suggest a reduced DNA repair capacity. Thus, we could assume that the DNA of subjects who developed PCa would be more susceptible to oxidative stress (Figure 6) and would have more potential alterations [58]. This observation corroborates the drop in glutamic acid, which is a component of glutathione involved in antioxidant defense. In addition, just like glutamic acid, 2-hydroxyadenine serves as fuel for the Krebs cycle (TCA). 7-keto cholesterol is metabolically linked to two other discriminating metabolites: sphinganine and sphingosine, known to be related to lysosomal disease [59]. The levels of these three metabolites are lower in men who developed PCa during the follow-up. These three metabolites are involved in the synthesis of sphingolipids and are found more abundantly in healthy subjects. At this point, it can be hypothesized that males who develop PCa will have increased synthesis of ceramides/sphingolipids. This observation would be in agreement with the reported role of sphingolipids in prostatic carcinogenesis [60,61,62], and also the previous work on the role of cholesterol and its derivatives in the biogenesis of membranes in PCa [63]. A hypothesis is that sphingolipids represent a potent class of apoptosis regulators in cancer cells [64]. Another hypothesis is that these metabolites could be implicated in the inflammatory process as it has been already observed in cancer [65]. This latter hypothesis could also explain the increased plasma level of eicosadienoic acid (EPA) in males who develop PCa [66]. Knowing that these three discriminating metabolites participate in the same metabolic pathway, we can envisage a major role for ceramides/sphingolipids in cancer carcinogenesis. These results are in agreement with the lipidomic disorders previously described in PCa patients [67].

Tiglyl carnitine belongs to the carnitine metabolic pathway. Carnitine is bio-synthesized from lysine and methionine, comprising a quaternary ammonium function. The main role of L-carnitine is to transport long-chain acyl groups of fatty acids in the mitochondrial matrix for the generation of ATP via beta-oxidation and TCA during the energetic catabolism of lipids. Its role is well documented, even in healthy patients, and in patients with metabolic disorders [68]. Previously, the NMR-based study had shown an increase in lysine [52]. This was linked to an increase in the synthesis of carnitine to support beta-oxidation. In addition, recent metabolomics studies show a protective effect of carnitines in people without prostate cancer due to an increase in serum levels [69]. Several studies have demonstrated the anti-inflammatory and antioxidant properties of acetyl-L-carnitine by “stabilizing” effects on the mitochondrial membrane. In our study, the decrease in plasma level of tiglyl carnitine was associated with PCa, which reinforces the idea of a “down-regulation” consumption of glutamic acid and 2-hydroxyadenine. This suggests an increase in the speed of the enzymatic reactions constituting the TCA in males who will develop PCa.

Serotonin activates the two interdependent MAP kinase and PI3K/Akt signaling pathways to induce proliferation, migration, and differentiation in PCa cell lines [70]. The PI3K pathway interferes with the activation of the mTOR pathway, which is activated by the accumulation of glutamine. In our study, the drop-in serotonin plasma levels in men who developed PCa during follow-up could indicate peripheral consumption or an “appetite” of pre-neoplastic prostate cells for this metabolite.

Ethyl oleate is an ester of oleic acid, the main monounsaturated fatty acid in the body. It is frequently associated with cholesterol esters and triglycerides. It is used for the synthesis of phospholipids but can also be oxidized to provide energy. In this study, the positive association between oleate and PCa risk as well as the negative association between 7-keto cholesterol and PCa risk suggest a massive use of the cholesterol ester. This results in the availability of oleate. In addition, operating in the “intense or increased” mode of TCA (as we have previously suggested) can reduce the energy production from fatty acids, allowing the accumulation of oleic acid in the plasma [67].

Phosphoric acid is also a majority anion in the intracellular compartment, and its accumulation can be linked to the expenditure of cellular energy (ATP => ADP + Pi). We have observed an increase in plasma orthophosphoric acid levels in men who developed prostate cancer during the follow-up. A first hypothesis would be an increase in phosphate as a consequence of the decreased levels of glutamic acid, carnitine, serotonin, aminopurine, and the ability to garner sources of phosphate for cell proliferation [55]. A second hypothesis would be that a disturbance in the calcium-phosphate balance could be involved. The role of citrate (previously identified in the NMR-based study from our group) in relation to calcium makes it possible to envisage the presence of “osteoblast-like” cells as described in prostate cancer [71]. In addition, an excess of phosphate is reported during disorders initiating carcinogenesis: gene instability, neovascularization, and cell toxicity [72].

To the best of our knowledge, this was the first study using a robust 12 min UHPLC-HRMS-based metabolomic analysis [73] to investigate the relationship between baseline plasma metabolites profiles and long-term prostate cancer risk in a large prospective male cohort.

The strengths of our study include the combination of a robust 12 min UHPLC-HRMS-based metabolomic assay with a large prospective cohort design and long follow-up, as well as a powerful and comprehensive statistical analysis with rigorous validation. Nevertheless, our study has several limitations. First, the age of males included in this study was mainly between 45–60 years, which may not fully represent the whole male population. Second, the metabolomic analysis was performed with a single blood draw, and, thus, the intra-individual variability of metabolic profiles over time was not controlled in this study. Despite that, several metabolomic studies showed good stability and reproducibility for most metabolites [74,75]. Moreover, further validation of these selected metabolites will be necessary by using a targeted quantitative analysis in an independent prospective cohort.

In conclusion, this prospective study revealed several early metabolic markers, identified by UHPLC-HRMS based plasma untargeted metabolomic profiles, that were associated with the risk of developing PCa within the following decade. Our results support dysregulation of amino acids and sphingolipid metabolism in males who subsequently developed PCa. After validation in other independent prospective studies, our study may contribute to (1) the development of new prevention and early screening strategies to identify males with a high risk of PCa well before symptoms appear, and (2) a better understanding of the etiology of this complex disease.

## Figures and Tables

**Table 1:** Characteristics of prostate cancer cases and matched controls.

Characteristics	Controls (*n* = 272)		Cases (*n* = 146)		
	Mean/N	SD/%	Mean/N	SD/%	*p*-Value *
Age at baseline (years)	54.3	4.6	54.7	4.8	0.09
Age at baseline (categories)					/
<45 years	6	2.2	3	2.1	
≥45–<50 years	53	19.5	27	18.5	
≥50–<55 years	71	26.1	38	26.0	
≥55–<60 years	113	41.5	62	42.5	
≥60–<65 years	29	10.7	16	11.0	
Age at diagnosis (years)	63.0	5.0	/	/	/
Time between blood collection and diagnosis (years)	8.3	3.0	/	/	/
Gleason ≥ 7 **	62	42.5	/	/	/
BMI (kg/m^2^)	25.0	3.0	25.4	3.0	0.07
BMI (categories)					/
Underweight (<18.5 kg/m^2^)	2	0.7	1	0.7	
Normal weight (≥18.5–<25 kg/m^2^)	133	48.9	72	49.3	
Overweight (>25 kg/m^2^)	137	50.4	73	50.0	
Season of blood draw					/
March–May (Spring)	102	37.5	56	38.4	
October–November (Fall)	36	13.2	21	14.4	
December–January (Winter)	134	49.3	69	47.3	
Smoking status					/
Non smokers	242	89.0	130	89.0	
Smokers	30	11.0	16	11.0	
SU.VI.MAX intervention group					/
Supplementation	123	45.2	65	44.5	
Placebo	149	54.8	81	55.5	
Family history of prostate cancer					0.01
No	261	96.0	130	89.0	
Yes	11	4.0	16	11.0	
Prostate-specific antigen (ng/mL)	1.3	1.2	3.4	3.4	<0.0001
Prostate-specific antigen (categories)					<0.0001
<3 ng/mL	256	94.1	97	66.4	
≥3 ng/mL	16	5.9	49	33.6	
Physical activity					0.7
Irregular	68	25.0	32	21.9	
<1 h walk or equivalent	57	21.0	35	24.0	
≥1 h walk or equivalent	147	54.0	79	54.1	
Educational level					0.9
Primary school	63	23.2	31	21.2	
Secondary school	101	37.1	56	38.4	
≥High-school degree	108	39.7	59	40.4	
Alcohol intake (g/day)	29.8	22.5	26.9	21.4	0.1

* *p*-value from conditional logistic regression models. ** missing for *n* = 10. Age (categories), BMI (categories), smoking status, season of blood draw, and SU.VI.MAX intervention group were the matching factors between cases and controls. BMI, body mass index; SD, standard deviation.

## Data Availability

Raw MS data analyzed in this study will be openly available in the EMBL-EBI MetaboLights data repository (MetaboLights; http://www.ebi.ac.uk/metabolights (accessed on 18 June 2021)) with the identifier MTBLS2783.

## Supplementary Materials

The following are available online at https://www.mdpi.com/article/10.3390/cancers13133140/s1, Figure S1: Score plot of OPLS-DA model for the Discovery cohort, Figure S2: AU-ROC for the OPLS-DA model validation, Figure S3: Score plot of PCA with QC samples (highlighted), Table S1: Internal Standards (ISs) and concentration, Table S2: Important metabolites in the discrimination of participants who developed PCa during the follow-up from controls selected by sPLS-DA.

## Abbreviations

AU-ROC, area under the receiver operating characteristic curve; BMI, body mass index; CI, confidence interval; FC, fold change; FDR, false discovery rate; IS, internal standard; LASSO, least absolute shrinkage and selection operator; NMR, nuclear magnetic resonance; ns, not significant; OR, odds ratio; OPLS-DA, orthogonal partial least squares discriminant analysis; PCa, prostate cancer; PCA, principal component analysis; PSA, prostate-specific antigen; Rt, retention time (in minutes); SU.VI.MAX, *Supplémentation en Vitamines et Minéraux Antioxydants*; SD, standard deviation; sPLS-DA, sparse partial least squares discriminant analysis; UHPLC-HRMS, ultra-high-performance liquid chromatography-high resolution mass spectrometry; VIP, variable importance in the projection.
