# Supplementary material for: Plasma Metabolomics for Discovery of Early Metabolic Markers of Prostate Cancer Based on Ultra-High-Performance Liquid Chromatography-High Resolution Mass Spectrometry
Source: Cancers (Basel). 2021 Jun 23;13(13):3140. doi: 10.3390/cancers13133140 (PMC8268247; doi:10.3390/cancers13133140)
Supplement: Supplementary file 1 [file cancers-13-03140-s001.zip › cancers-1178576-SI.pdf]

**Reagents and Chemicals used for the samples preparation and the UHPLC-HRMS Metabolomic Analysis**

HPLC grade solvent (acetonitrile, methanol) and formic acid were purchased from Merck KGaA (Darmstadt, Germany). Ultrapure water ($H_2O$) was collected from a Milli-Q system (Millipore, Billerica, MA). Internal Standards (ISs), Carnitine C8:0-d3, Carnitine C16:0-d3, FFA C18:0-d3, CA-d4, CDCA-d5, Phe-d5, and Trp-d5 were purchased from Cambridge Isotope (Tewksbury, MA); FFA C16:0-d3 was purchased from C/D/N Isotopes Inc. (Pointe-Claire, Québec), and LPC 19:0 was supplied by Avanti Polar Lipids (Alabaster, AL).

**Supplemental Table S1**. Internal Standards (ISs) and concentration.

| Internal Standards (ISs) | Concentration in extraction solvent (ug/mL) |
|---|---|
| Carnitine C2:0-d3 | 0.16 |
| Carnitine C10:0-d3 | 0.10 |
| Carnitine C16:0-d3 | 0.15 |
| LPC 19:0 | 0.75 |
| FFA C16:0-d3 | 2.50 |
| FFA C18:0-d3 | 2.50 |
| CA-d4 | 1.85 |
| CDCA-d4 | 1.49 |
| Leu-d3 | 5.00 |
| Val-d8 | 4.00 |
| Phe-d5 | 3.61 |
| Trp-d5 | 4.25 |

**Supplemental Table S2**. Important metabolites in the discrimination of participants who developed PCa during the follow-up from controls selected by sPLS-DA.

| Metabolites | m/z | Rt | FC | p-value | Adj. p-value | FDR |
|---|---|---|---|---|---|---|
| Eicosadienoic acid | 309.2776 | 6.04 | 0.18 | 1.43E-05 | 3.07E-03 | 4.38E-04 |
| Ethyl oleate | 311.2928 | 6.97 | 0.41 | 5.12E-05 | 1.10E-02 | 1.37E-03 |
| Phosphate | 98.9843 | 0.41 | 0.09 | 1.02E-05 | 2.19E-03 | 4.38E-04 |
| Tiglyl carnitine | 244.1539 | 1.31 | -0.10 | 6.31E-04 | 1.35E-01 | 1.35E-02 |
| L-glutamic acid | 148.0609 | 0.38 | -0.19 | 6.75E-11 | 1.44E-08 | 7.22E-09 |
| Serotonin | 177.1022 | 0.48 | -0.25 | 1.30E-08 | 2.78E-06 | 6.96E-07 |
| 2-hydroxyadenine | 152.0567 | 0.39 | -0.24 | 8.77E-10 | 1.88E-07 | 6.25E-08 |
| 7-Keto cholesterol | 401.3407 | 5.96 | -0.10 | 1.01E-04 | 2.16E-02 | 2.40E-03 |
| Sphinganine | 302.3046 | 3.64 | -0.33 | 8.88E-16 | 1.90E-13 | 1.90E-13 |
| Sphingosine | 300.2902 | 3.51 | -0.21 | 1.21E-05 | 2.60E-03 | 4.33E-04 |

m/z: detected mass; Rt, retention time (in minutes); FC, fold change; FDR, false discovery rate; p-value were computed by using Multiple Experiment Viewer (MeV_4_9_0_r2731_win); Adj. p-value, p-value with Bonferroni adjustment.

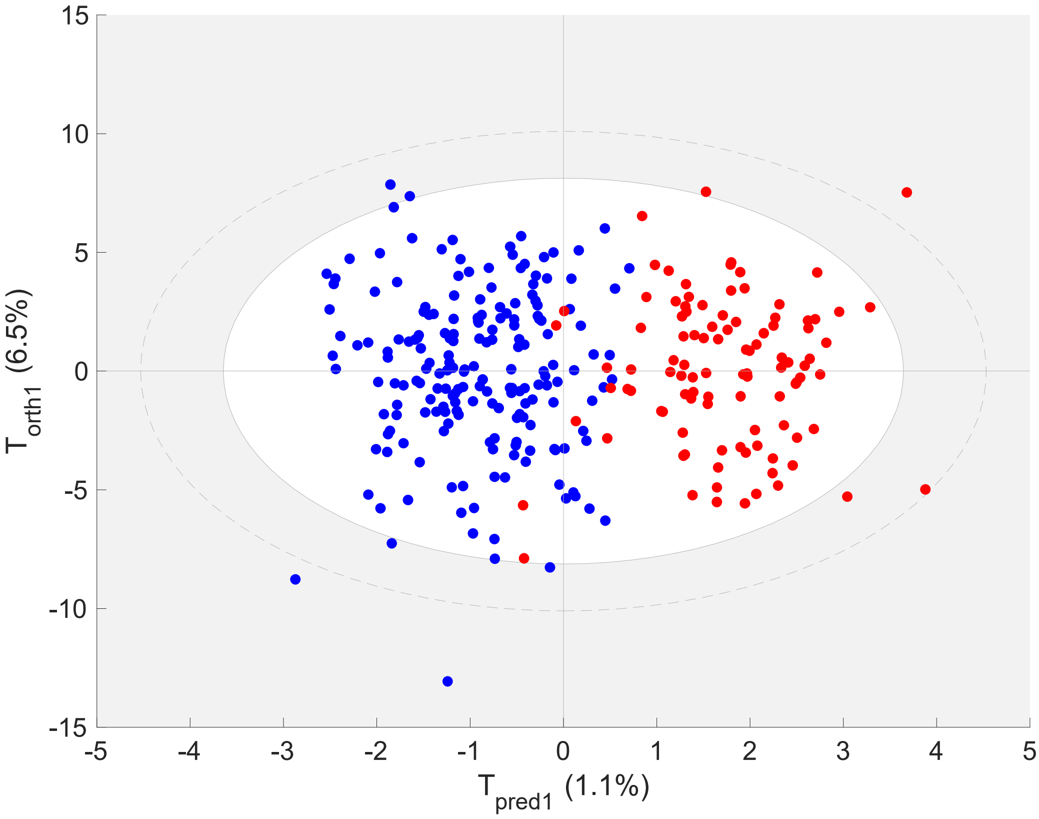


**Supplemental Figure S1**. Score plot of OPLS-DA model for the Discovery cohort.

Prostate cancer cases (n=102, red circle), matched controls (n=190, blue circle).

Components: 1(predictive) + 4(orthogonal); R2: 0.73, Q2cum: 0.48.

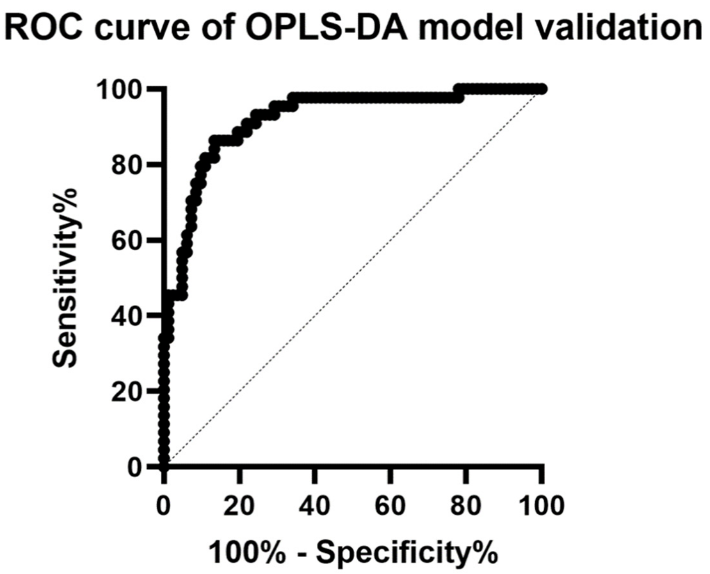


**Supplemental Figure S2**. AU-ROC for the OPLS-DA model validation.

The OPLS-DA model from the discovery cohort was validated by prediction of samples in the validation cohort (remaining 30% of all samples, PCa cases: n= 44 / Controls: n= 82). AU-ROC: 0.92 (Sensitivity: 86.36%; Specificity: 86.59%), 95% confidence interval (0.87, 0.97), P value: < 0.0001.

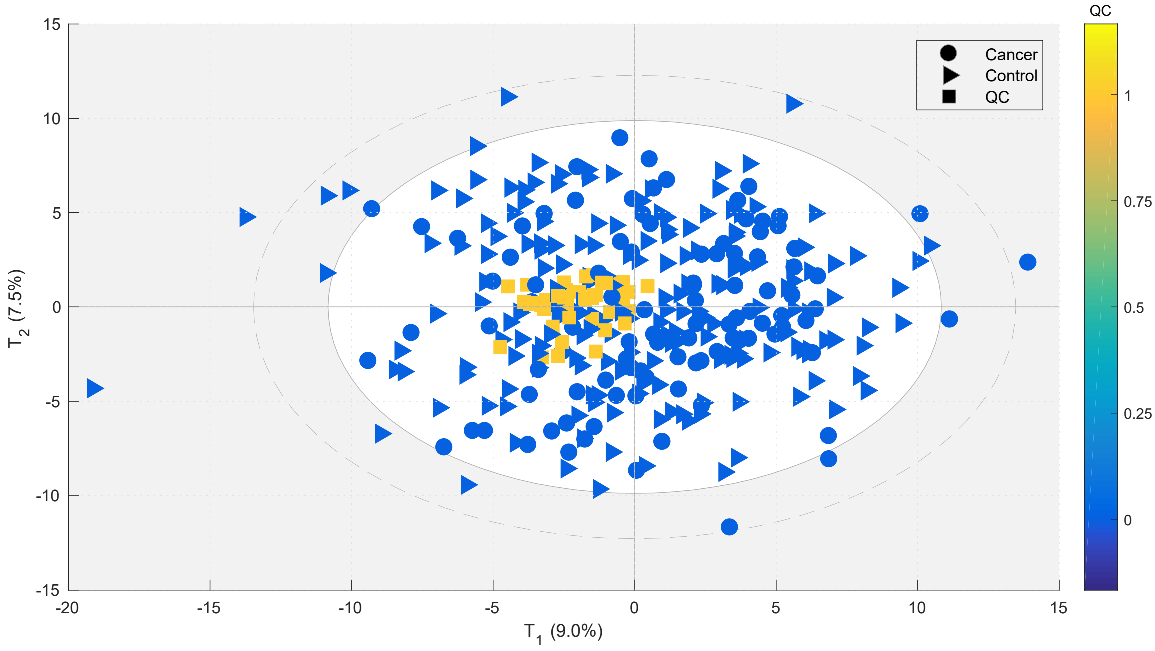


**Supplemental Figure S3**. Score plot of PCA with QC samples (highlighted).
